# The Werther effect: adherence of Italian newspapers to the “reporting on suicide” recommendations

**DOI:** 10.1093/eurpub/ckac131.483

**Published:** 2022-10-25

**Authors:** G Scaioli, G Giacomini, P Galvagno, F Bert, G Lo Moro, R Siliquini

**Affiliations:** 1 Department of Public Health, University of Turin, Turin, Italy; 2 A.O.U. City of Health and Science of Turin, Turin, Italy

## Abstract

**Background:**

Reporting a case of suicide on a newspaper could lead to an emulation effect (Werther effect). It is important to report suicide cases by following specific recommendations implemented by World Health Organization (WHO). This study aimed to analyze articles published on Italian newspapers to quantify the adherence to the WHO recommendations in suicide report.

**Methods:**

All the articles published in the three most important Italian newspapers from June 2019 to May 2020 that described one or more cases of suicide were included. Two researchers analyzed all the articles through an ad-hoc checklist, constructed on the basis of the WHO recommendations, that included 18 “negative” items (e.g. “presence of suicide-related words in the title”) and nine “positive” items (e.g. “the article reports the contacts of a suicide-prevention hotline”). For each negative item a “-1” point, and for each positive item a + 1 point, was assigned. Multivariable linear regressions were performed to identify factors related with a lower adherence to the WHO recommendations and with higher social engagement of the articles.

**Results:**

A total of 110 articles were analyzed. In the 73% of the cases, the individual was male. The 14.5% of the suicide cases were homicide-suicide, while 9% were femicide-suicide. The median score of the checklist was -6 (IQR 3). Only 5% of the articles had at least one positive item. The word “suicide” (or related words) were present in 90% of the titles. Multivariable analyses showed that female suicides were associated with a higher score of the checklist (coeff 0.816, p = 0.039). No correlation was retrieved between the score of the checklist and the social media engagement of the articles included.

**Conclusions:**

Italian newspapers do not follow WHO recommendations on how to report suicide cases, leading to a potential emulation effect. Public health professionals should raise decision makers’ and journalists’ awareness about the importance of these recommendations.

**Key messages:**

• Since Italian newspapers are not compliant with the recommendations of the World Health Organization on how to report a suicide case, there is the risk of a potential emulation effect.

• Specific training for journalists and other professionals in the field of communication should be implemented, to raise the awareness on the importance of the “reporting on suicide” recommendation.

